# Disturbance Increases Microbial Community Diversity and Production in Marine Sediments

**DOI:** 10.3389/fmicb.2016.01950

**Published:** 2016-12-02

**Authors:** Pierre E. Galand, Sabrina Lucas, Sonja K. Fagervold, Erwan Peru, Audrey M. Pruski, Gilles Vétion, Christine Dupuy, Katell Guizien

**Affiliations:** ^1^Sorbonne Universités, UPMC Univ Paris 06, CNRS, Laboratoire d’Ecogéochimie des Environnements Benthiques (LECOB), Observatoire OcéanologiqueBanyuls sur Mer, France; ^2^UMR 7266 Littoral, Environnement et Sociétés, Institut du littoral et de l’environnement, CNRS – Université de La RochelleLa Rochelle, France

**Keywords:** diversity, metagenome, marine sediment, succession, disturbance, ecology, oxygen, RNA

## Abstract

Disturbance strongly impacts patterns of community diversity, yet the shape of the diversity-disturbance relationship remains a matter of debate. The topic has been of interest in theoretical ecology for decades as it has practical implications for the understanding of ecosystem services in nature. One of these processes is the remineralization of organic matter by microorganisms in coastal marine sediments, which are periodically impacted by disturbances across the sediment-water interface. Here we set up an experiment to test the hypothesis that disturbance impacts microbial diversity and function during the anaerobic degradation of organic matter in coastal sediments. We show that during the first 3 weeks of the experiment, disturbance increased both microbial production, derived from the increase in microbial abundance, and diversity of the active fraction of the community. Both community diversity and phylogenetic diversity increased, which suggests that disturbance promoted the cohabitation of ecologically different microorganisms. Metagenome analysis also showed that disturbance increased the relative abundance of genes diagnostic of metabolism associated with the sequential anaerobic degradation of organic matter. However, community composition was not impacted in a systematic way and changed over time. In nature, we can hypothesize that moderate storm disturbances, which impact coastal sediments, promote diverse, and productive communities. These events, rather than altering the decomposition of organic matter, may increase the substrate turnover and, ultimately, remineralization rates.

## Introduction

One of the key features of microbial communities in most natural ecosystems is that they harbor a tremendous diversity (Keller and Zengler, 2004). Community diversity is affected by a number of biotic interactions but also by abiotic factors such as the surrounding environmental conditions. Environmental conditions change with time and can fluctuate dramatically when impacted by episodic disturbances. A disturbance can be defined as “any relatively discrete event in time that disrupts ecosystem, community, or population structure and changes resources, substrate availability, or the physical environment” (Pickett and White, 1985). The response of communities to disturbance has been a central interest in ecology studies for decades. More specifically, investigations have aimed to resolve how disturbances in an ecosystem influence the diversity of the communities (Sousa, 1984). In this respect, the intermediate disturbance hypothesis (IDH; Grime, 1973; Connell, 1978) suggests that the relationship between diversity and disturbance can be represented graphically as a unimodal distribution. Community diversity is low when disturbance levels are low and increases with disturbance frequency or strength. If disturbance persists, however, diversity decreases again. Nevertheless, recent studies have refuted the idea of a positive relationship between disturbance and community diversity. A survey of empirical studies reported that non-significant relationships were the most common (Mackey and Currie, 2001), and a recent paper proposed, based on both empirical and theoretical grounds, that the IDH should be abandoned (Fox, 2013). For microorganisms, soil experiments showed that diversity declined with increasing disturbance frequencies, (Kim et al., 2013) and a negative diversity-disturbance relationship was also observed in natural marine sediments (Boer et al., 2009).

Recently, it has been emphasized to incorporate genetic diversity into disturbance-diversity studies because the topic remains a major knowledge gap (Banks et al., 2013). Despite the recognition of the importance of genetic diversity in ecology (Hughes et al., 2008), its role as a driver for microbial diversity and function remains poorly understood. Overall, data on the IDH for microorganisms remains scarce because studies have mainly focused on the impact of disturbance on community composition, rather than diversity (Allison and Martiny, 2008; Berga et al., 2012; Yeo et al., 2013). Communities are hypothesized to be resistant or resilient to disturbance when they are not definitely altered in their composition (Allison and Martiny, 2008).

Episodic disturbances, which are thought to have an important role in maintaining diversity, impact many of the ecosystems of the Earth. Coastal sediments are regularly exposed to disturbances due to increases in turbulence that result from the dissipation of energetic flows in the benthic boundary layer. These disturbances occur with different strengths and at different temporal scales. Tidal flow generates periodic increases of turbulence above the sediment with moderate intensity, whereas waves cause larger but more sporadic turbulence increase, which lead to sediment resuspension. Turbulence intensity in the benthic boundary layer controls diffusive fluxes across the sediment-water interface; as a consequence, turbulence intensity fluctuations disturb the stratification of dissolved compounds in the sediments and may affect organic matter degradation (Arzayus and Canuel, 2005).

Organic matter degradation is an essential service provided by heterotrophic microorganisms (Ducklow, 2008). This is particularly true in estuarine sediments and deltaic systems, where most of the terrestrial organic matter delivered by rivers is deposited before being remineralized or buried (Hedges et al., 1997; Burdige, 2007). Understanding the fate of terrestrial or marine organic matter exported down to sediments may help gain greater insight into the cycling of carbon on a global scale and its possible alteration by climate change and anthropogenic perturbations (Regnier et al., 2013; Reichstein et al., 2013). Degradation or remineralization of the complex mixture of molecules composing sediment organic matter is mediated by the combined activity of an array of both aerobic and anaerobic microorganisms with different metabolic potential. Although aerobic processes are more effective, the anoxic degradation rates are often similar to the oxic rates in surface sediments (Arndt et al., 2013). The identity of the main players in the terminal degradation process and the structure of the communities involved are not always well known. The composition of the microbial communities can for instance be correlated to the origin and lability of organic matter (Fagervold et al., 2014), the geochemical composition of the sediments (Jorgensen et al., 2012) and pigment concentrations (Bienhold et al., 2012). The impact of disturbance, which may disrupt the commonly observed vertical zonation of marine sediments, remains poorly studied.

Here, we test the hypothesis that disturbance impacts microbial community diversity in coastal sediments during organic matter mineralization. We conducted an experiment in which sediment cores were regularly exposed to enhanced turbulence at the sediment-water interface, without sediment resuspension, mimicking the effect of moderate swell events. Sediments were enriched with plant-derived detritus to simulate the sedimentation of labile terrestrial organic matter. Organic matter degradation was monitored during the course of the experiment and so was the concentration of oxygen. Microbial abundance was measured as a way to derive production. Bacterial and archaeal communities were described before and after each disturbance event at two sediment depths by targeting both 16S rRNA genes and 16S rRNA transcripts to identify the active fraction of the communities. We also constructed metagenomes to test whether disturbance impacted potential sedimentary metabolic pathways and to verify whether possible changes in community taxonomic composition were correlated with changes in functional gene composition.

## Materials and Methods

### Experimental Setup

A total of 36 sediment cores enriched with duckweed (*Lemna* sp.) were placed in a water tank incubated in the dark at 16°C (see “Supporting Information” for details; **Supplementary Table S1**). The sediment cores were exposed to disturbance in the form of a spatially uniform diffusive turbulence generated by an oscillating grid that controlled the diffusive fluxes across the sediment-water interface [**Supplementary Figure S1** (Lucas et al., 2016)]. Turbulence frequency and strength were chosen to simulate the disturbance generated by moderate swell events in the infralittoral area. Overall, the experiment included four disturbance events preceded by calm periods. Twenty-four-hour disturbance events (turbulent velocity = 6.15 cm s^-1^) were separated by calm intervals of 10 days (turbulence < 1.18 cm s^-1^). The entire experiment lasted for 5 weeks. The low turbulence in the calm periods corresponded to turbulent conditions in the benthic boundary layer during usual wind-driven circulation. The disturbance events did not cause any sediment resuspension.

Sampling was conducted before and after each disturbance event. A total of eight different time points (T0–T7) were sampled (four before disturbance and four after disturbance). For each sampling, three cores were removed from the water tank. The cores were sliced and sediment samples were taken from two layers: 0–5 mm (surface) and 5–10 mm (deep). Organic matter concentrations and microbial abundances were measured on triplicate cores, and oxygen, fatty acids, and microbial molecular analysis were conducted on one of the three cores.

### Fatty Acids, Organic Matter, Oxygen, and Bacterial Abundance

Labile OM compounds, namely total carbohydrates, was assessed using colorimetric methods according to previously published protocols (Dubois et al., 1956; Barnes and Blackstock, 1973; Bradford, 1976). Total fatty acids were extracted by direct acid transmethylation and analyzed using a Saturn 2100T iontrap GC–MS instrument (VARIAN, Les Ulis, France) equipped with a fused-silica-capillary column (Factor Four, VF-Waxms, 30 m × 0.25 mm ID, 0.25 mm film thickness, VARIAN). Details of the extraction procedure and analytical conditions are given in Bourgeois et al. (2011). Oxygen concentrations were profiled at a 200 μm vertical spatial resolution every 15 min with a Unisense O_2_ Clark-type microsensor (Unisense, Aarhus, Denmark) from 20 mm above to 10 mm below the sediment-water interface. Time series of the average O_2_ concentration in the sediment layers (0–5 mm, 5–10 mm) were calculated from these profiles.

Prokaryotic abundance was measured by flow cytometry. Five milliliters of sediment was fixed with 0.2 μm filtered formaldehyde solution (vol/vol, 2% final concentration), and cells were separated from the sediment and homogenized according to the protocol described by (Lavergne et al., 2014). One milliliter of sediment was diluted sequentially to 1/2000 with 0.01 M sodium pyrophosphate (NaPp) and was mixed by vortexing. Samples were then incubated at + 4°C for 30 min before sonication (60 W for 30 s). Finally, an aliquot of the sample was stained with SYBR Green I (1:10,000) for 15 min in the dark and was analyzed by flow cytometry as previously described (Lavergne et al., 2014). Bacterial production was derived from the increase in bacterial abundance and was expressed as the number of cells per mL of sediment and per day. Production was calculated for each time point as: Production(T_2_) = (ln(Ab(T_2_)) - ln(Ab(T_1_)))/(T_2_-T_1_), where Ab is the prokaryotic abundance and T is the time.

### Nucleic Acid Extraction, RT-PCR, and Sequencing

DNA and RNA were co-extracted using the RNA PowerSoil Total RNA Isolation Kit and the DNA Elution Accessory Kit (MoBio, Carlsbad, CA, USA) from 1 g of frozen sediment according to the manufacturer’s protocol. RNA samples were DNAse treated using the RNase-Free DNase Set (Qiagen, Valencia, CA, USA) and concentrated with the RNeasy MinElute Cleanup Kit (Qiagen). The RNA were reverse-transcribed with random primers using the SuperScript III Reverse Transcriptase kit (Invitrogen, Carlsbad, CA, USA) and tested for the presence of contaminating genomic DNA by performing PCR before the reverse transcription.

Portions of the 16S rRNA genes were amplified with the bacterial primer pair 27F (5′-AGRGTTTGATCMTGGCTCAG-3′) and 519R (5′-GTNTTACNGCGGCKGCTG-3′), and archaeal primers 349F (5′-GYGCASCAGKCGMGAAW-3′) and 806r (5′-GGACTACVSGGGTATCTAAT-3′). A 30 cycle PCR with the HotStarTaq Plus Master Mix Kit (Qiagen, Valencia, CA, USA) was conducted under the following conditions: 94°C for 3 min, followed by 28 cycles of 94°C for 30 s, 53°C (bacteria), or 50°C (archaea) for 40 s and 72°C for 1 min, after which a final elongation step at 72°C for 5 min was performed. Pyrosequencing was conducted by a commercial laboratory (Research and Testing Laboratory, Lubbock, TX, USA) on a Roche 454 FLX (Brandford, CT, USA) using commercially prepared Titanium reagents. Sequences have been deposited to the GenBank Sequence Read archive under number SAMN04126915–SAMN04126946.

For the metagenomes, libraries for shotgun sequencing were prepared using Nextera DNA Sample Preparation Kit (Illumina, San Diego, CA, USA) with 50 ng of DNA from each sample. Library insert size was determined by Experion Automated Electrophoresis Station (Bio-Rad, Hercules, CA, USA). The insert size of the libraries ranged from 300 to 850 bp (average 500 bp). Pooled libraries (12 pM) were loaded to a 600 Cycles v3 Reagent cartridge (Illumina) and the sequencing was performed on Miseq (Illumina) by a commercial laboratory (Research and Testing Laboratory, Lubbock, TX, USA). Metagenomes are archived in MG-RAST under accession numbers 4612993.3, 4612994.3, 4612995.3, 4612996.3, and 4612997.3.

### Sequence Analysis

All reads that had a mismatch with the 16S rRNA primers, contained ambiguous nucleotides (N) or were longer than 390 after the forward primer, were removed. Then, reads that had ≥3% of bases with Phred values <27 (0.2% per-base error probability) were removed. These steps are recommended to ensure that when clustering at 97%, the influence of erroneous reads is minimized (Huse et al., 2010). Sequences were clustered at a 97% threshold using Uclust (Edgar, 2010). Representative sequences of each operational taxonomic unit (OTU) were classified by comparison with Blast to the Greengenes database (DeSantis et al., 2006). Sequence analyses were conducted with Pyrotagger (Kunin and Hugenholtz, 2010). To compare bacterial communities for diversity analysis, all samples were randomly re-sampled to the size of the sample containing the fewest sequences (*n* = 950 for Bacteria and *n* = 424 for Archaea) using Daisy Chopper (Gilbert et al., 2009).

For metagenomes, sequence ends were cut to remove adapters. Sequences were considered low quality when >5 bases had a phred score <15 and were subsequently removed. FastqJoin was used to merge paired end reads when possible with a minimum overlap setting of 8 bp and a maximum difference of 10%. Both paired and unpaired sequences were kept for automated annotation in MG-RAST using Hierarchical Classification subsystems against the KEGG Orthology database. Annotations were conducted with a maximum *e*-value cutoff of 1e^-10^, a minimum 60% identity cutoff and 15 amino acids as the minimum alignment length cutoff. Identified microbial gene families (specified by KEGG Orthology groups) were grouped into metabolic pathways (**Supplementary Table S2**). Metagenome reads were normalized by evenly resampling at random to 1,254,500 annotated reads per sample to allow comparison.

### Data Analysis

Bacterial diversity was estimated for both the RNA and DNA fractions by calculating the Shannon diversity index (H′) and by the standardized effect size (SES; Kembel et al., 2010), which is a standardized measure of phylogenetic diversity. Phylogenetic diversity was computed using the Picante package (Kembel et al., 2010) in R. To calculate SES, 300-bp-long representative sequences for each OTU were aligned using MUSCLE (Edgar, 2004) and the alignment was then cleaned to remove non-overlapping sequence regions. A phylogenetic tree was constructed using FASTTREE (Price et al., 2010). The observed phylogenetic diversity was compared to the average phylogenetic diversity in a randomly generated community (null model) and divided by the standard deviation of phylogenetic distances in the null model (Webb et al., 2008). The null model randomizes community data matrix with the independent swap algorithm maintaining species occurrence frequency and sample species richness (Kembel, 2009). SES is equivalent to -1 times the Nearest Relative Index (NRI; Webb et al., 2002). Faith’s PD (Faith, 1992) is the most common measure of phylogenetic diversity, but because the number of taxa in a sample affects PD and because the number of taxa varied significantly between our samples, we chose to use SES instead. Positive SES values indicate greater phylogenetic distance among co-occurring species than expected by chance, whereas negative values indicate small phylogenetic distance.

The Bray-Curtis similarity index was computed to compare the community and functional gene composition between samples and to conduct cluster analysis. The effects of disturbance and time on community composition were tested with a permutational multivariate analysis of variance using distance matrices as implemented in the adonis function in the Vegan package (v.2.3-0) in the R software (v.3.1.2). To identify OTUs that had a significant difference in abundance between groups we used an ANOVA with a Tukey–Kramer *post hoc* test as implemented in STAMP (Parks et al., 2014).

## Results

### Dynamics of Oxygen, Carbohydrates, and Fatty Acid Concentrations

**FIGURE 1:**
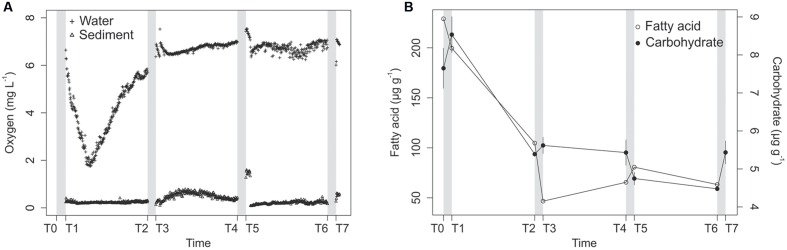
**Oxygen concentrations in the water (crosses) and sediments (triangles) along the course of the experiment** **(A)**, and Lemna sp. fatty acids (open circles) and total carbohydrate concentrations (filled circles) in the experimental sediments **(B)**. Lemna sp. fatty acids are represented by the sum of four characteristic saturated, monounsaturated, and polyunsaturated fatty acids (see “Material and Methods”). Gray bars represent the disturbance events.

Each of the four disturbance events, which lasted for 24 h (T0–T1, T2–T3, T4–T5, and T6–T7), oxygenated the water above the sediment, resulting in O_2_ saturation. Oxygen concentrations in the overlying water decreased at the beginning of each interleaved calm interval (T1–T2, T3–T4, and T5–T6; **Figure 1A**). Oxygen concentrations in the sediment did not follow the same trend as in water: the concentration increased after the first disturbance and remained low at 0.20 mg L^-1^ on average in the upper layer of the sediments during the following calm interval (T1–T2; **Figure 1A**). After the second disturbance event (T2–T3), the oxygen concentrations in the upper part of the sediment increased with a peak of 0.60 mg L^-1^ 3 days after the end of the disturbance, and then decreased to 0.40 mg L^-1^ at the end of the calm interval (T3–T4; **Figure 1A**). The third disturbance (T4–T5) immediately increased oxygen concentrations up to 1.50 mg L^-1^ in the upper sediment layer, but the concentrations rapidly dropped to lower levels than during the first and second calm intervals (**Figure 1**). The last disturbance (T6–T7) increased the oxygen concentration in the sediment to 0.50 mg L^-1^.

In summary, turbulent disturbance oxygenated the mesocosm water, thereby increasing oxygen flux to the sediment. However, the average concentrations in the sediment varied during the course of the experiment, and the 0–5 mm surficial sedimentary layer remained anoxic on average (<0.6 mg L^-1^), with the exception of the first hours after the T4–T5 disturbance, during which the sediment was in hypoxia. The deeper part of the sediment (5–10 mm) was always anoxic (data not shown).

The average carbohydrate concentration was 8.1 μg g^-1^ in the surface sediment layer at the beginning of the experiment and decreased to 5.4 μg g^-1^ at T2 (**Figure 1B**). The concentrations remained low, with values varying from 4.5 to 5.6 μg g^-1^ of dry sediment. Among the fatty acids quantified by GC–MS, four were characteristic of the fatty acid signature of the duckweed used to enriched the sediments (C16:0, C18:1ω9 *cis*, C18:2ω6 *cis*, and C18:3ω3) and accounted for 77% of the fatty acids in the sediments at T0. The concentrations of these biomarkers decreased strongly from T0 to T3 and remained at low concentrations after that (**Figure 1B**), which indicates that the decrease in carbohydrate concentrations reflected the degradation of duckweed detritus in the surface sediment layers.

### Prokaryote Production and Community Diversity

**FIGURE 2:**
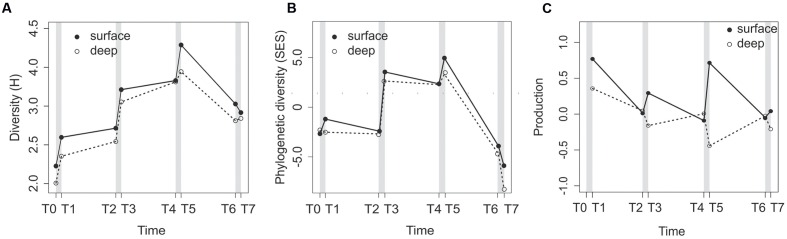
**Changes in the RNA-based community diversity** **(A)** and phylogenetic diversity **(B)**, and prokaryote production expressed as the change in the number of cells per mL of sediment and per day **(C)** in the surface and deep sediment layers along the course of the experiment. Gray bars represent the disturbance events.

Bacterial community diversity, represented by the Shannon index (H), varied during the course of the experiment in both the surface and deep layers (**Figure 2A**) and for both the RNA and DNA fractions (**Supplementary Figure S2**). For the RNA fraction, the surface and deep sediment bacterial communities had similar patterns (**Figure 2A**). Bacterial community diversity increased sharply after each of the three first disturbance events and remained stable or even decreased during calm periods (**Figure 2A**). For the DNA fraction, the surface and deep layers had similar diversity dynamics patterns, with the exception of the T2–T3 disturbance event (**Supplementary Figure S2**), but the patterns differed from the RNA fraction. The first disturbance resulted in a sharp decrease in bacterial diversity (**Supplementary Figure S2**). Then, increases were observed with turbulence, and there was a decrease at the end of the experiment.

Similar to community diversity, the phylogenetic diversity for the RNA fraction, measured as SES, increased in surface sediments after each of the first three disturbance events (**Figure 2B**) and decreased after T5. Moreover, the SES values were negative at T0, T1, and T2 for both sediment depths (**Figure 2B**), positive in the T3–T5 samples, and then negative again in the T6 and T7 samples. The deep sediment SES patterns were similar to the surface patterns. All SES values were significant against a null model, with the exception of the surface sample at T1.

In surface sediments (0–5 mm), the prokaryote production, expressed as the number of cells per mL of sediment per day, increased after each disturbance event and decreased during calm intervals (**Figure 2C**). In deep sediments (5–10 mm), we observed an opposite trend, with a decrease in prokaryote production after each turbulence events (**Figure 2C**).

### Effect of Time and Disturbance on Community Composition

**FIGURE 3:**
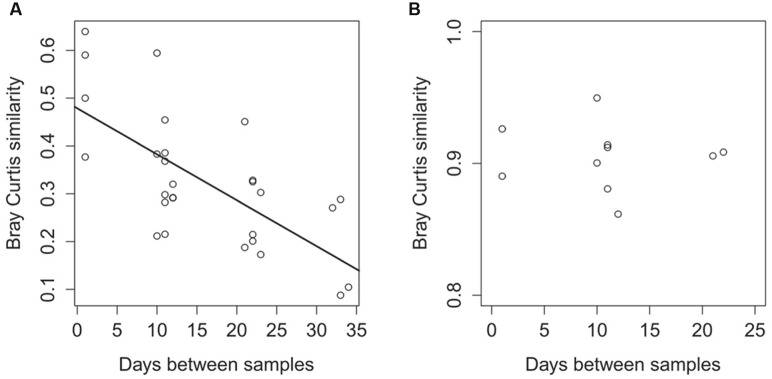
**Differences in the taxonomic community composition between samples** **(A)** and differences in the functional community composition **(B)** in relation to the time separating the samples. Differences in the community composition are calculated with the Bray-Curtis similarity index based on 16S rRNA sequences for the taxonomic comparison **(A)** and are based on the functional genes from the metagenomes annotated against the KEGG Orthology database **(B)**.

We calculated the Bray-Curtis similarity index between each possible sample pair to compare community composition similarity with time. In both surface and deep sediment samples, the Bray-Curtis values were higher when samples were separated by shorter time intervals (**Figure 3A**), and the similarity decreased as the time separating communities increased (ANOVA, *F*_1,26_ = 22.76, *p* < 0.001), which indicated that turbulence (samples separated by 1 day) had less effect on community composition compared to time.

We further tested the effect of disturbance and time with a permutational multivariate analysis of variance using distance matrices (adonis statistics). Disturbance did not impact the community composition at the RNA level (*F*_1,8_ = 0.04, *p* = 0.73), whereas time was a significant factor impacting the community composition (*F* = 0.36, *p* = 0.001). There was no difference in community composition between depths (*F* = 0.046, *p* = 0.65).

### Community Succession

**FIGURE 4:**
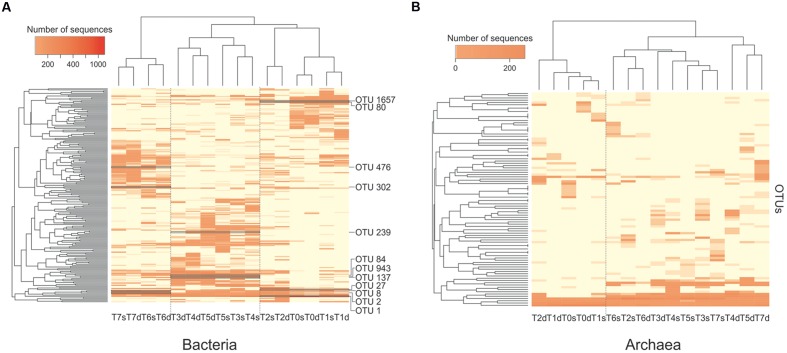
**Hierarchical clustering of the composition of the bacterial** **(A)** and archaeal **(B)** communities based on 16S rRNA transcripts sampled along the course of the experiment (T0–T7) from the surface (s) and deep (d) layers of the sediment. The heatmap shows the relative abundance of each operational taxonomic unit (OTU) (rows). The names of the bacterial OTUs that were found to characterize each of the three main clusters of samples (column) are indicated.

The composition of microbial communities was compared between samples with a hierarchical cluster analysis based on the Bray-Curtis index. Active bacteria communities (RNA) were separated into three main clusters (**Figure 4A**). One contained samples taken at T0, T1, and T2, one contained samples taken at T3, T4, and T5 and the last contained samples from T6 to T7. At T0, T1, T2, T6, and T7, surface and deep sediment communities grouped together, showing similar community composition between depths. At T3 and T5, the deep and surface communities were separated, suggesting a stronger stratification of the communities with depth (**Figure 4A**). Pre- and post-disturbance samples grouped together, indicating that disturbance events had a lower impact on the composition of the active communities than calm periods, despite the increase of community diversity. The second disturbance event represented an exception because it induced a major change in community composition (**Figure 4A**). The T5 and T6 communities were separated in two different clusters, which indicated that the third calm interval had a strong impact on the active bacterial community composition.

**FIGURE 5:**
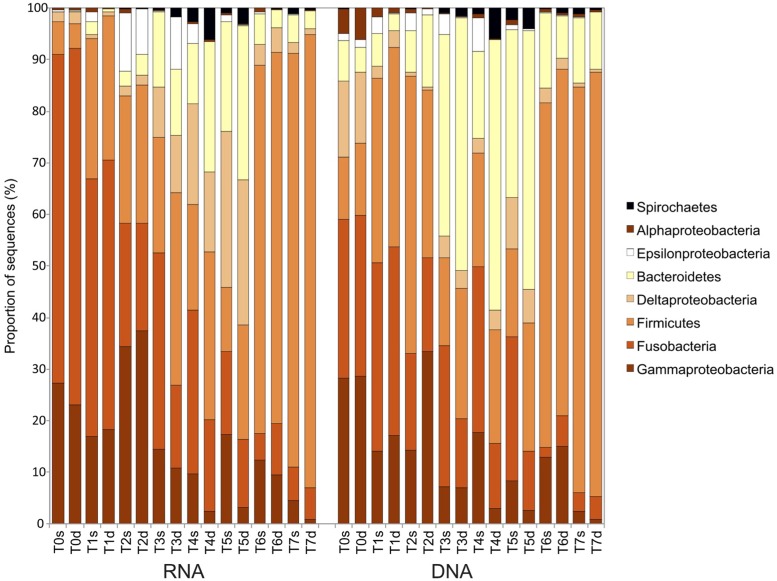
**Taxonomic affiliation and proportion of the bacterial 16S rRNA sequences in sediments sampled during the course of the experiment (T0–T7) in the surface (s) and deep (d) layers for both the 16S rRNA transcripts (RNA) and the 16S rRNA genes (DNA)**.

At the phylum/class level, the first cluster of active bacteria (RNA fraction; T0–T2) contained a majority of *Gammaproteobacteria*, *Fusobacteria*, and *Firmicutes* sequences (**Figure 5**). In the second cluster (T3–T5), the proportion of *Deltaproteobacteria* and *Bacteroidetes* sequences increased. The third cluster (T6 and T7) was composed mostly of *Firmicutes* (>70% of the sequences). The composition of the bacterial communities described with DNA was similar to that observed at the RNA level, but there were more *Bacteroidetes* sequences and fewer *Deltaproteobacteria*.

At the OTU level, the first cluster (T0–T2) had a higher abundance of sequences belonging to OTU 1 and OTU 1657 (**Figure 4A**; **Supplementary Figure S3**), which were similar to sequences from *Propionigenium maris* (*Fusobacteria*; 98% similarity), an anaerobic strain found in sediments that can ferment glucose and succinate primarily to propionate (Watson et al., 2000). Other specific OTUs included OTU 80, 98% similar to the sediment sequences belonging to the order *Clostridiales* (*Firmicutes*), and OTU 27, identified as *Vibrio kanaloae* (*Gammaproteobacteria*; 99% similarity), which can ferment different compounds as the sole carbon source (Thompson et al., 2003). The second cluster (T3–T5) had more sequences belonging to OTU 239, OTU 943, OTU 137, and OTU 84 (**Figure 4A**; **Supplementary Figure S3**). All belonged to the order *Desulfobacterales* (*Deltaproteobacteria*), a group of sulfate-reducing bacteria. The third cluster (T6 and T7) was characterized by the presence of OTU 476, OTU 302, OTU 8, and OTU 2, all belonging to *Clostridiales* (*Firmicutes*), an order with many fermenting bacteria.

For Archaea, active communities (RNA fraction) were separated in two main clusters (**Figure 4B**). The first contained samples from T0 and T1, and the second cluster grouped the other samples, with the exception of the deep T2 sample. At the phylum/class level, the T0–T1 cluster contained mostly *Thaumarchaeota* (>85% of the sequences), whereas the second cluster (T2–T6) contained mostly *Methanomicrobia* sequences (**Supplementary Figure S4**). At the DNA level, the *Methanomicrobia* were less abundant, and there were more sequences from the SAGMEG-1 and C2 group, as well as *Methanobacteria*.

At the OTU level, the T0–T1 cluster had a higher abundance of sequences belonging to OTU 54, OTU 3, and OTU 4 (**Supplementary Figure S5**), which were 99, 98, and 97% similar to the *Candidatus Nitrosopumilus koreensis* AR1 (*Thaumarchaeota*), respectively (Park et al., 2012). The second cluster (T2–T6) was characterized by OTU 8, which was 99% similar to the methanogen *Methanococcoides methylutens (Methanomicrobia)*, and OTU 13 and OTU 711, which were 99 and 97% similar, respectively, to *Methanolobus oregonensis (Methanomicrobia)*. All belong to the family Methanosarcinaceae, a group known for performing all pathways of methanogenesis.

### Functional Genes Dynamics

We constructed metagenomes for surface sediment samples T2–T6 and obtained a total of ca. 66.8 million sequences after quality check (**Supplementary Table S3**). The Bray-Curtis index, calculated from the abundance of annotated sequences found in the functional genes categories, showed that there was no correlation between the time separating two samples and the functional gene composition of the samples (ANOVA, *F* = 0.03, *p* = 0.85; **Figure 3B**).

Within the annotated metagenome sequences, we identified the level two gene categories that varied the most between samples (**Supplementary Figure S6**). The number of sequences associated with membrane transport was highest at T2 and then decreased to similar lower levels. Energy metabolisms showed an opposite trend. There were fewer sequences at T2, and after an increase at T3, the number of sequences did not change significantly. Sequences for genes involved in cell motility were abundant in samples T2 and T4 and then decreased at T5 and again at T6. Cell growth and death increased after each turbulence event.

**FIGURE 6:**
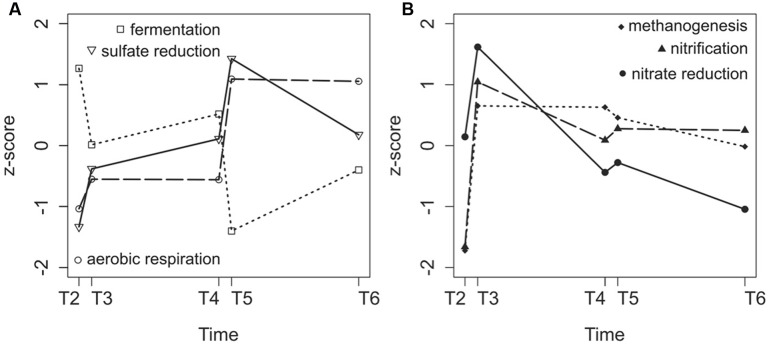
**Variation in fermentation, sulfate reduction and aerobic respiration gene sequence numbers** **(A)** and methanogenesis, nitrification and nitrate reduction gene numbers **(B)** expressed as *z*-scores during the course of the experiment (T2–T6). Sequence counts were obtained after annotating metagenomes against the KEGG Orthology database.

We then specifically targeted some metabolic pathways present in sediments that may be impacted by disturbances. Genes typical for methanogenesis, sulfate reduction, nitrate reduction, aerobic respiration, fermentation, and nitrification were identified (**Supplementary Table S2**). The genes that had the highest number of sequences were chosen to monitor the pathway dynamics in the sediments (**Supplementary Table S2**). All of the gene sequences increased in abundance after the first turbulence event, with the exception of genes involved in fermentation (**Figure 6**). After the second turbulence event, genes for sulfate reduction and aerobic respiration showed large increases (**Figure 6A**), whereas genes for nitrate reduction and nitrification showed small increases (**Figure 6B**). The number of sequences associated with sulfate reduction and aerobic respiration increased overall over time during the course of the experiment, whereas the number of sequences for genes associated with methanogenesis, nitrification, and nitrate reduction decreased after the first turbulence event (**Figure 6**).

To compare the metagenome and amplicon results, we counted the number of 16S rRNA amplicon sequences identified as methanogens (belonging to the classes *Methanobacteria*, *Methanococci*, and *Methanomicrobia*) and sulfate reducers (order *Desulfobacterales*). For methanogens, the DNA and RNA counts showed similar patterns, which indicated that the microbes that were present were also active. The disturbance events resulted in an increase of the number of methanogen 16S rRNA sequences. For sulfate reducers, the DNA and RNA patterns were also similar (**Supplementary Figure S7**). The second and third disturbance events resulted in an increase in the number of sequences in a pattern similar to that observed for metagenomic functional genes (**Figure 6**).

## Discussion

We showed that disturbance increased the diversity of marine sediment bacterial communities during the first 3 weeks of incubation in a mesocosm experiment. Our finding can be interpreted in the context of the IDH, IDH (Grime, 1973; Connell, 1978), which predicts that disturbance will increase community diversity up to a certain level of disturbance strength or frequency, after which diversity will decrease. The increase in diversity is hypothesized to originate from the creation of niches because disturbances contribute to environmental heterogeneity. These niches are colonized by new species and the effect of competitive dominance is reduced (Violle et al., 2010). If disturbance is too frequent, extinction will occur, only the few species able to cope with an unstable environment will grow, and diversity will decrease. In our experiment, diversity increased until T5 (3 weeks) and then started to decrease. The last disturbance event did not augment diversity, and we propose that we observed the decrease predicted by the IDH. The IDH has been challenged recently, however, and the shape of the diversity-disturbance relationship remains a matter of discussion (Mackey and Currie, 2001; Randall Hughes et al., 2007; Fox, 2013). This is the first report of a positive disturbance-diversity relationship for benthic microorganisms. The hypothesis was tested previously with *in situ* data from marine sediment, where depth was used as a proxy for disturbance (Boer et al., 2009), but only a weak relationship was observed. A number of studies have been conducted on soil microorganisms, in which no relationship (Fierer et al., 2003; Wakelin et al., 2010; Deng et al., 2011) or only a negative impact of disturbance has been reported (Kim et al., 2013). Here, the positive relationship was observed both at the beginning of the experiment, when the nutrient load was high, and later, when the organic carbohydrate concentrations were significantly lower. This is an important point because community diversity may be directly influenced by the resources available in the ecosystem. It has been shown, for instance, that high nutrient availability may reduce the number of species (Torsvik et al., 2002) and that the decline in energy over time may impact the level of diversity (Scholes et al., 2005). Our observation of diversity increasing independent of nutrient availability argues for a direct effect of disturbance on diversity. It should, however, be noted that the availability of electron acceptors is also very important in determining sediment community composition. The differences in cell numbers observed between surface and deep layer may reflect differences in oxygen availability between depths.

Interestingly, phylogenetic diversity was also positively impacted by disturbance, which indicates that disturbance promoted the co-occurrence of phylogenetically distinct species. Bacteria with distant common ancestors are more likely to be ecologically different. Thus, the presence of distantly related species after disturbance supports the notion that diversity increased because additional niches were created and occupied by ecologically different species, which reduced competitive exclusion. Inversely, the low values measured toward the end of the experiment indicate phylogenetic clustering, which can be interpreted as communities structured by environmental filtering (Webb et al., 2002). The environment selects a subset of ecologically similar taxa able to dominate under the given environmental conditions.

We also showed that in surface sediments, diversity bursts were accompanied by an increase in prokaryote production. The finding of stimulated production is supported by the observation of a relative increase, after each disturbance event, of genes that belonged to the “cell growth and death” category, which may indicate that cells were actively growing. The link between production and diversity is a key topic in ecology (Naeem et al., 2009; Cardinale et al., 2012) and a positive relationship is usually observed for community diversity (Gillman and Wright, 2006; Cardinale et al., 2012). For marine microbes, contrasting results show a negative relationship for bacterial richness (Reinthaler et al., 2005; Obernosterer et al., 2010) but a positive relationship between production and the phylogenetic diversity of active bacteria (Galand et al., 2015). High-diversity communities may use resources more efficiently through positive interactions and/or niche partitioning (complementary effect); alternatively, more diverse communities are more likely to contain highly productive organisms (sampling effect; Loreau and Hector, 2001). In our experiment, the relationship did not hold in the deeper layer of the sediments, where production decreased with disturbance. Negative relationships between production and diversity have been shown for bacteria under controlled experimental settings (Horn et al., 2003; Becker et al., 2012), suggesting that other factors may constrain the relationship. We also found that prokaryote production decreased during calm intervals. Whereas increased microbial abundance has been detected at intermediate disturbance level in soil (Kim et al., 2013), our data suggest that high production was not maintained after disturbance events, or that only a few of the sediment species could maintain growth as time passed.

We identified specific groups of active microorganisms that increased in relative gene abundance when the microbial biomass increased. Methanogens and sulfate reducers both increased in sequence abundance with disturbance and may be contributors to the overall biomass bursts. The question remains as to why these groups were stimulated and others, such as fermenters, were inhibited. We can speculate that disturbance made fermentation end products more available and that they were then used by methanogens and sulfate reducers, which both represent the final step of anaerobic organic matter remineralization.

Although we observed changes in sequence abundance for some groups, our experiment showed that disturbance did not have a systematic impact on the composition of active communities, which indicates that the communities overall changed according to the time of incubation. The presence of a temporally dynamic community implies that communities will not recover to their initial composition after disturbance. In such a dynamic ecosystem, resilience can probably not be observed. Our results suggest that for defining a general response to disturbance in terms of community composition (Allison and Martiny, 2008) an ecosystem has to be stable or in a steady state. However, stability, which results from a combination of biotic and abiotic factors, is never or rarely observed in coastal sediments impacted by seasonal variations in physical forcing and nutrient loads.

We identified three main stages during our incubation that corresponded to different community compositions. The first period was dominated by sequences affiliated with potential fermenting bacteria and with archaea identified as ammonia oxidizers. During the second stage, we detected more sequences belonging to sulfate-reducing bacteria and methanogenic archaea. For the last stage, methanogens were still present, and sequences associated with potential fermenters, different from the first ones, appeared. We hypothesize that the general pattern of changing communities over time followed the transformation of organic matter contained in the sediments.

The overall potential microbial metabolism did not change as much as the composition of the communities during the course of the incubation. The first stage was the most dissimilar, with relatively fewer genes associated with energy metabolism but more membrane transport, whereas cell motility decreased toward the end of the incubation. However, the comparison of the gene function composition did not show significant changes with time, which indicated that there was a certain level of functional redundancy within the communities. Assemblies that appear different at the 16S rRNA gene level may actually contain similar metabolic functions. These functions would be maintained during the course of the experiment, but the identity of the microorganisms bearing these functions would change with the evolving environmental conditions. More studies are needed to resolve the question of microbial functional redundancy because our observation remains limited to one type of experimental microbial ecosystem.

## Conclusion

We showed that disturbance had a positive effect on sediment microbial communities by increasing their production and both their community and phylogenetic diversity during the first 3 weeks of the experiment. This result implies that disturbance stimulated the overall community growth and promoted the development of many phylogenetically different microorganisms. These diverse communities occupy a wide range of niches and consume a variety of substrates produced during the sequential anaerobic degradation of organic matter. In nature, we can hypothesize that the moderate storm disturbances that regularly impact coastal sediments also promote diverse communities. These events, rather than disturbing the decomposition of organic matter, probably increase the substrate turnover and, ultimately, remineralization rates.

## Author Contributions

SL and KG designed the experiments; PG, SL, AP, EP, SF, GV, CD, and KG carried out the work; SL, PG, and KG interpreted the results; PG wrote the manuscript that was revised and improved by all the coauthors

## Conflict of Interest Statement

The authors declare that the research was conducted in the absence of any commercial or financial relationships that could be construed as a potential conflict of interest.
