# Supplementary material for: Disturbance Increases Microbial Community Diversity and Production in Marine Sediments
Source: Front Microbiol. 2016 Dec 2;7:1950. doi: 10.3389/fmicb.2016.01950 (PMC5133735; doi:10.3389/fmicb.2016.01950)
Supplement: Supplementary file 1 [file Data_Sheet_1.docx]

**Detailed experimental setup**

Sediment cores were sampled by scuba divers in the Bay of Banyuls sur Mer, North West Mediterranean Sea, at a depth of 35 m in May 2012. The first 2 cm of the sediment was then sieved through a 200 μm mesh. The sieved sediments were composed of fine particles (d_50_=45 μm) and had an organic matter content of 2.27 %. Sediments were used to build 8x30 cm cores (Supplementary Figure 1) that were left for 1 month in aquaria in the dark for the sediment to settle and to let a redox gradient form. Sediments were then enriched with duckweed (*Lemna sp*.) to simulate the input of terrigenous organic matter observed in coastal areas. Cleaned and freeze-dried duckweed gathered earlier from a local river was ground to a fine powder and incorporated into the first 2 cm of the sediment cores to reach a final 5 % organic matter content (Supplementary Table 1).

Sediment cores enriched were placed in a water tank (0.60 × 0.60 × 0.51 m height), and the sediment-water interface of all cores was adjusted to the same height (Supplementary Figure 1). The mesocosm was incubated in the dark at 16 °C.

The sediment cores were exposed to disturbance in the form of a spatially uniform diffusive turbulence generated by an oscillating grid that controlled the diffusive fluxes across the sediment-water interface (Supplementary Figure 1). The grid was formed of crossed square bars with a 4 cm opening placed horizontally above the sediment cores. The assembly oscillated vertically and was driven by a motor rotating at a set frequency and stroke length. The intensity of the turbulence at the sediment surface was set by varying the motor frequency, stroke length, and/or the distance between the grid position and the surface of the sediment. Turbulence frequency and strength were chosen to simulate the disturbance generated by moderate swell events in the infralittoral area. Overall, the experiment included four disturbance events preceded by calm-periods. Twenty-four-hour disturbance events (turbulent velocity=6.15 cm s^-1^), which recreated swell events, were separated by calm-intervals of 10 days (turbulence < 1.18 cm s^-1^). The low turbulence in the calm-periods corresponded to turbulent conditions in the benthic boundary layer during usual wind-driven circulation. The disturbance events did not cause any sediment resuspension.

Table S1. Composition of the Banyuls sur Mer Bay sediments, duckweed and the experimental sediment after duckweed enrichment.

| Sediment composition  (mg g^-1^) | Bay sediments | Duckweed | Experimental sediments  (0-5 mm, T0) |
| --- | --- | --- | --- |
| Organic matter | 22.7 | 910 ±0.5% | 47.9 ±8% |
| Hydrocarbon | 2.7 | 482 ±1.2% | 5.4 ±22.3% |
| Proteins | 1.2 | 283 ±2.8% | 3.4 ±4.6% |
| Lipids | 0.26 | 88 ±3.2% | 1.04 ±3.9% |
| Labil organic matter | 18.3 % | 93.7% | 29.2% |

Table S2. Relative abundances of functional genes involved in selected metabolic pathways. The genes used to monitor pathway dynamics in the sediment cores are marked in bold.

| Gene | KO | Number of sequences | | | | |
| --- | --- | --- | --- | --- | --- | --- |
|  |  | T2 | T3 | T4 | T5 | T6 |
| Methanogenesis |  |  |  |  |  |  |
| **hdrA; heterodisulfide reductase subunit A [EC:1.8.98.1]** | **K03388** | **1411** | **3823** | **3800** | **3626** | **3144** |
| hdrB; heterodisulfide reductase subunit B [EC:1.8.98.1] | K03389 | 173 | 192 | 282 | 230 | 278 |
| hdrC; heterodisulfide reductase subunit C [EC:1.8.98.1] | K03390 | 93 | 135 | 135 | 110 | 180 |
| hdrD; heterodisulfide reductase subunit D [EC:1.8.98.1] | K08264 | 3 | 10 | 8 | 4 | 0 |
| hdrE; heterodisulfide reductase subunit E [EC:1.8.98.1] | K08265 | 0 | 0 | 3 | 3 | 0 |
| Sulfate reduction |  |  |  |  |  |  |
| dsrA; sulfite reductase, dissimilatory-type alpha subunit [EC:1.8.99.3] | K11180 | 67 | 231 | 268 | 322 | 176 |
| dsrB; sulfite reductase, dissimilatory-type beta subunit [EC:1.8.99.3] | K11181 | 88 | 208 | 230 | 356 | 189 |
| **aprA; adenylylsulfate reductase, subunit A [EC:1.8.99.2]** | **K00394** | **437** | **636** | **739** | **1014** | **753** |
| aprB; adenylylsulfate reductase, subunit B [EC:1.8.99.2] | K00395 | 103 | 177 | 203 | 186 | 161 |
| Nitrate reduction |  |  |  |  |  |  |
| narG; nitrate reductase 1, alpha subunit [EC:1.7.99.4] | K00370 | 727 | 451 | 363 | 569 | 686 |
| narH; nitrate reductase 1, beta subunit [EC:1.7.99.4] | K00371 | 296 | 246 | 184 | 229 | 267 |
| narJ; nitrate reductase 1, delta subunit | K00373 | 74 | 29 | 24 | 17 | 37 |
| narI; nitrate reductase 1, gamma subunit [EC:1.7.99.4] | K00374 | 90 | 104 | 122 | 86 | 109 |
| **napA; periplasmic nitrate reductase NapA [EC:1.7.99.4]** | **K02567** | **1829** | **2359** | **1619** | **1677** | **1401** |
| napB; cytochrome c-type protein NapB | K02568 | 109 | 50 | 33 | 22 | 40 |
| nirB; nitrite reductase (NAD(P)H) large subunit [EC:1.7.1.4] | K00362 | 965 | 599 | 581 | 645 | 610 |
| nirD; nitrite reductase (NAD(P)H) small subunit [EC:1.7.1.4] | K00363 | 82 | 63 | 55 | 45 | 54 |
| Fermentation |  |  |  |  |  |  |
| ldhA; D-lactate dehydrogenase [EC:1.1.1.28] | K03778 | 596 | 290 | 397 | 249 | 460 |
| LDH, ldh; L-lactate dehydrogenase [EC:1.1.1.27] | K00016 | 225 | 156 | 172 | 130 | 327 |
| **adhE; acetaldehyde dehydrogenase / alcohol dehydrogenase [EC:1.2.1.10 1.1.1.1]** | **K04072** | **1854** | **1496** | **1640** | **1092** | **1378** |
| Nitrification |  |  |  |  |  |  |
| **hao; hydroxylamine oxidase [EC:1.7.3.4]** | **K10535** | **97** | **298** | **227** | **241** | **239** |

Table S3. Sequence characteristics of the 5 metagenomes constructed from experimental sediments.

| MG-RAST ID | Metagenome Name | Sequence Count | Post QC | Number of sequences annotated* |
| --- | --- | --- | --- | --- |
| 4612997.3 | 29D | 9,373,858 | 8,052,168 | 1,254,500 |
| 4612996.3 | 27D | 25,423,363 | 17,257,086 | 1,571,742 |
| 4612995.3 | 25D | 24,768,866 | 19,468,764 | 2,938,279 |
| 4612994.3 | 23D | 13,978,721 | 11,193,843 | 1,694,639 |
| 4612993.3 | 21D | 13,224,240 | 10,844,799 | 1,807,191 |


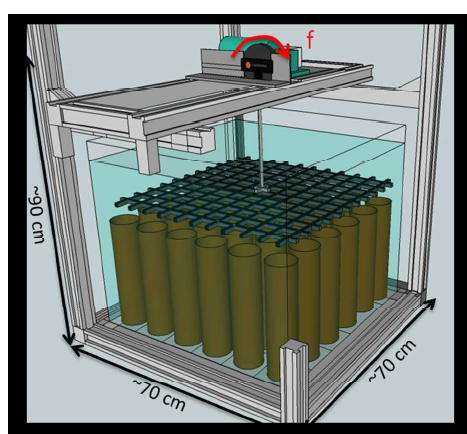


Figure S1. Experimental design with oscillating grid above sediment cores incubated in a mesocosm. F represents the frequency of the engine moving the grid. The figure is reproduced with permission from : Lucas, S., Moulin, F., and Guizien, K. (2016). Oscillating grid mesocosm for studying oxygen dynamics under controlled unsteady turbulence. Limnol. Oceanogr. Meth. 14(1) : 1-13.

Figure S2. Changes in the DNA-based community diversity expressed as Shannon index (H) in the surface and deep sediment layers along the course of the experiment.

Figure S3. Relative proportion of the bacterial OTUs identified as characteristic of the different times of incubation.

Figure S4. Taxonomic affiliation and proportion of the archaeal 16S rRNA sequences in sediments sampled during the course of the experiment (T0-T7) in the surface (s) and deep (d) layers for both the 16S rRNA transcripts (RNA) and the 16S rRNA genes (DNA).

Figure S5. Relative proportion of the archaeal OTUs identified as characteristic of the different times of incubation.

Figure S6. Number of sequences of the level 2 gene categories within the annotated metagenome sequences that varied the most between samples.

Figure S7. Number of 16S rRNA sequences belonging to methanogenic archaea (left) and sulfate reducing bacteria (right) for both the 16S rRNA transcripts (RNA) and the 16S rRNA genes (DNA) during the course of the incubation.
